# Remotely Delivered Nonpharmacologic Treatments of Chronic Pain: Insights From Embedded Pragmatic Clinical Trials

**DOI:** 10.1093/geroni/igaf122.2123

**Published:** 2025-12-31

**Authors:** Kushang Patel, M Carrington Reid

## Abstract

Chronic pain increases with advancing age and contributes to decreased functioning, reduced quality of life, and high healthcare costs. Given the limited efficacy of analgesic medications and greater risk of adverse effects with advancing age, clinical practice guidelines recommend nonpharmacologic treatments. While evidence for nonpharmacologic pain treatments in older adults is accumulating, access to and uptake of nonpharmacologic treatments remain limited, particularly among rural-dwelling and minoritized populations. In the proposed symposium, we will report findings from 4 NIH-funded pragmatic clinical trials that are evaluating the implementation and effectiveness of remotely delivered nonpharmacologic treatments for chronic pain among middle-aged and older adults in health systems across the United States. First, Eric Roseen will present key challenges and solutions to implementing remotely delivered tai chi for knee osteoarthritis in 4 health systems in 4 geographic regions. Second, Elise Hoffman will present the adaptation and implementation of a telehealth delivered nurse care management program, involving care coordination, cognitive-behavioral therapy, and tele-exercise for rural primary care patients who have chronic pain. Third, Natalia Morone will present findings from a telehealth delivered, group-based mindfulness intervention in primary care patients with chronic low back pain. Fourth, Kushang Patel will report on age differences in the effects of remotely delivered self-paced and group-based mindfulness interventions (versus usual care) among Veterans who have chronic pain. Lastly, Cary Reid will deliver a critical discussion of the research findings and provide recommendations for future embedded pragmatic clinical trials, highlighting special considerations for improving pain management in older primary care patients.

